# Widespread *Bradyrhizobium* distribution of diverse Type III effectors that trigger legume nodulation in the absence of Nod factor

**DOI:** 10.1038/s41396-023-01458-1

**Published:** 2023-06-24

**Authors:** Alicia Camuel, Albin Teulet, Mélanie Carcagno, Fazal Haq, Valérie Pacquit, Djamel Gully, Marjorie Pervent, Clémence Chaintreuil, Joël Fardoux, Natasha Horta-Araujo, Shin Okazaki, Safirah Tasa Nerves Ratu, Fatou Gueye, Jerri Zilli, Nico Nouwen, Jean-François Arrighi, Haiwei Luo, Peter Mergaert, Laurent Deslandes, Eric Giraud

**Affiliations:** 1 IRD, Laboratoire des Symbioses Tropicales et Méditerranéennes (LSTM), UMR IRD/Institut Agro/INRAE/Université de Montpellier/CIRAD, TA-A82/J– Campus de Baillarguet 34398, Montpellier cedex 5, France; 2 PHIM Plant Health Institute, Université de Montpellier, IRD, CIRAD, INRAE, Institut Agro, Montpellier, France; 3 University of Cambridge, Sainsbury Laboratory (SLCU), Cambridge, CB2 1LR UK; 4 LIPME, Université de Toulouse, INRAE, CNRS, Castanet-Tolosan, France; 5 Université Paris-Saclay, CEA, CNRS, Institute for Integrative Biology of the Cell (I2BC), 91198 Gif-sur-Yvette, France; 6 Graduate School of Agriculture, Tokyo University of Agriculture and Technology, Tokyo, 183-8509 Japan; 7 Carrefour International, Bureau Régional Afrique de l’Ouest, Dakar, Sénégal; 8 Embrapa Agrobiologia, Bairro Ecologia, Seropedica, Rio de Janeiro Brazil; 9 School of Life Sciences and State Key Laboratory of Agrobiotechnology, The Chinese University of Hong Kong, Hong Kong SAR, China

**Keywords:** Bacterial genetics, Plant ecology

## Abstract

The establishment of the rhizobium-legume symbiosis is generally based on plant perception of Nod factors (NFs) synthesized by the bacteria. However, some *Bradyrhizobium* strains can nodulate certain legume species, such as *Aeschynomene* spp. or *Glycine max*, independently of NFs, and via two different processes that are distinguished by the necessity or not of a type III secretion system (T3SS). ErnA is the first known type III effector (T3E) triggering nodulation in *Aeschynomene indica*. In this study, a collection of 196 sequenced *Bradyrhizobium* strains was tested on *A. indica*. Only strains belonging to the photosynthetic supergroup can develop a NF-T3SS-independent symbiosis, while the ability to use a T3SS-dependent process is found in multiple supergroups. Of these, 14 strains lacking *ernA* were tested by mutagenesis to identify new T3Es triggering nodulation. We discovered a novel T3E, Sup3, a putative SUMO-protease without similarity to ErnA. Its mutation in *Bradyrhizobium* strains NAS96.2 and WSM1744 abolishes nodulation and its introduction in an *ernA* mutant of strain ORS3257 restores nodulation. Moreover, ectopic expression of *sup3* in *A. indica* roots led to the formation of spontaneous nodules. We also report three other new T3Es, Ubi1, Ubi2 and Ubi3, which each contribute to the nodulation capacity of strain LMTR13. These T3Es have no homology to known proteins but share with ErnA three motifs necessary for ErnA activity. Together, our results highlight an unsuspected distribution and diversity of T3Es within the *Bradyrhizobium* genus that may contribute to their symbiotic efficiency by participating in triggering legume nodulation.

## Introduction

Nature has the remarkable capacity to evolve different pathways to reach the same phenotype. This is the case for the signaling cascade initiating the rhizobium-legume symbiosis, which leads to the formation of a new plant organ, the nodule, where the bacteria fix nitrogen for the plant’s benefit and receive in exchange photosynthates and other nutrients. The most common way to achieve the formation of this symbiotic organ involves flavonoids on the plant side and Nod factors (NFs) on the rhizobial side [1, 2]. This NF-dependent pathway has long been considered as a universal paradigm governing all rhizobium-legume symbioses. However, two other alternative pathways have been described during the last two decades [3–5]. The first one has been observed in some tropical legume species of the *Aeschynomene* genus that are nodulated by photosynthetic bradyrhizobia lacking the canonical *nod* genes required for NF synthesis [3]. The second was initially identified in *Glycine max*, a legume species known to normally require NFs to initiate symbiosis [4]. It has been shown that the cultivar Enrei can still be nodulated by a *nod* mutant of the non-photosynthetic strain *Bradyrhizobium elkanii* USDA61 [4]. This second alternative process differs to the first one in that this interaction relies on the functioning of a type III secretion system (T3SS), such secretory machinery being absent in most photosynthetic bradyrhizobia nodulating *Aeschynomene* [6]. In laboratory conditions, strain USDA61 as well as a large diversity of non-photosynthetic bradyrhizobia, can also nodulate *Aeschynomene indica* through their T3SS and independently of NFs [5]. This highlights the interest of *A. indica* to study these two alternative symbiotic processes.

The T3SS is a complex secretory machinery resembling a nanosyringe that is used by various Gram-negative bacteria to inject into the host cells type III effector proteins (T3Es) whose most common functions are to subvert host immunity [7, 8]. Rhizobial T3Es often act similarly as pathogenic T3Es and, depending on the host plant, can display opposing roles [9–11]. In some plants, they can repress the plant immune system and thus promote the infection and nodulation, whereas in other plants, they can be recognized by cytoplasmic plant receptors and trigger a strong plant immune response, called effector-triggered immunity (ETI), resulting in blockage of the rhizobial infection. Specific rhizobial T3Es can also circumvent the requirement of the NF signal and directly activate nodule formation. This is the case for the ErnA effector identified in *Bradyrhizobium vignae* strain ORS3257 [12]. This T3E, which is found in a wide range of *Bradyrhizobium* strains having a T3SS, plays a crucial role during the T3SS-dependent symbiosis with *Aeschynomene* [12]. Furthermore, ectopic expression of *ernA* in transgenic roots of *A. indica* triggers nodule-like structures in the absence of the bacteria [12]. The ErnA protein displays no homology with known functional domains, but it has been shown that ErnA targets the host nucleus and interacts with nucleic acids suggesting that ErnA could transcriptionally activate the nodulation program [12].

In the case of the T3SS-dependent symbiosis between *G. max* and *B. elkanii* USDA61, it is another T3E, named Bel2-5, which governs the interaction [13, 14]. Bel2-5 is also targeted to the host cell nucleus and contains a C-terminal small ubiquitin-like modifier (SUMO)-Protease domain shown to be required for the nodulation activity. This indicates that this T3E fulfills its role by deSUMOylation of plant protein targets. Taken together, these data indicate that different classes of rhizobial T3Es able to trigger nodulation and proposed to be called ET-Nods [15], use different mechanisms to activate the nodulation program in legumes.

In this study, we examined the ability of a collection of 196 *Bradyrhizobium* strains of various origins to use a NF-independent symbiotic process for nodulation of *A. indica*. We focused on genome-sequenced strains to establish correlations between genomic content and symbiotic properties. This led us to examine the following questions: are there phylogenetic groups distinct to photosynthetic bradyrhizobia able to develop a NF-independent, T3SS-independent symbiosis? Does the presence of ErnA in T3SS containing strains systematically confer the ability to develop a T3SS-dependent symbiosis? Can other T3Es distinct from ErnA act similarly and trigger nodulation in *A. indica*? Through a combination of comparative genomic analyses and mutagenesis of some candidate genes emerging from these analyses, we identified several novel ET-Nods and obtained more information about the ability of bradyrhizobia to nodulate legumes independently of NFs.

## Materials and methods

### Bacterial strains and growth conditions

All tested bacterial strains are listed in Supplementary Dataset S1. The *Bradyrhizobium* strains were grown in YM or AG medium at 28 °C [16, 17]. All strains were checked for purity by streaking on agar plate and for identity by ITS sequencing as described [18]. *Escherichia coli* strains were grown at 37 °C in modified Lysogeny Broth (LB) medium [19]. *Agrobacterium rhizogenes* Arqua1 and *Agrobacterium tumefaciens* GV3101 were grown at 28 °C on AG medium and *Ralstonia pseudosolanacearum* was grown in rich BG medium [20]. When required, the media were supplemented with the appropriate antibiotics at the following concentrations: 50–100 µg/mL kanamycin, 20 µg/mL nalidixic acid, 20 µg/mL cefotaxime and 100–200 µg/mL spectinomycin.

### Genome sequencing and phylogenomic analysis

The genomes of strains ORS86, ORS111, STM3557, STM3561, STM3562, STM3566, LMG29515 and LMG09283 were sequenced by combining long read and short read sequencing technologies as previously described [21], assembled using the CulebrONT pipeline [22] and annotated using the MicroScope platform [23]. The other genomes downloaded from the NCBI or GenBank databases were annotated de novo by the MicroScope pipeline, and are also publicly available from the MicroScope platform (http://www.genoscope.cns.fr/agc/microscope). From all these genome sequences, a phylogenomic tree was constructed using IQ-tree v2.2.0.3 [24] based on a multi sequence alignment of 725 genes of the core genome shared by at least 98% of *Bradyrhizobium* strains, generated by PPanGGolin v1.2.63 [25]. The phylogeny was inferred from the resulting alignment under the model recommended by ModelFinder [26] and branch supports with ultrafast bootstrap were estimated from 100,000 iterations [27]. Final editing was carried out using iTOL (https://itol.embl.de/) [28].

### Prediction of T3E repertoires, search of SUMO-proteases and phylogenetic analysis

The search of T3Es was performed as previously described [12]. The search for SUMO-Proteases was performed by interrogating the precomputed InterProScan annotations hosted by the MicroScope platform for each genome, using the InterPro identifier (IPR003653) corresponding to the C-terminal catalytic domain of the ULP1 protease family. Protein alignments of ErnA and SUMO-Proteases were performed using ClustalW (http://www.clustal.org/omega/) [29] and phylogenetic analysis using IQ-tree as described above.

### Plasmid construction, mutagenesis and complementation

Standard molecular biology techniques were used for all cloning work. All constructions made in this study are listed in Supplementary Dataset S2, which also includes the primers and the cloning strategies. Briefly, the insertional mutants were obtained by single-crossing over. For this purpose, a 200 to 300-bp internal fragment of the target gene was amplified by polymerase chain reaction (PCR) and cloned into a derivative of the non-replicative plasmid pVO155 carrying an antibiotic resistance marker appropriate to the strain to be mutated (pVO155-npt2-GFP-npt2-Cefo for NAS96.2 and LMTR13 strains; pVO155-npt2-GFP-npt2-Sp for WSM1744 and ORS86) [5, 30]. The deletion WSM1744∆*sup3* mutant was obtained by double crossing over. For this, 750 to 1000-bp PCR fragments corresponding to the upstream and downstream flanking regions of the *sup3*_*WSM1744*_ gene (JAAVLW01_10281) were merged by overlap extension PCR and cloned into the pNPTS129 vector, which carries the *sacB* suicide gene [31]. Subsequently, a spectinomycin resistance cassette was introduced between the upstream and downstream flanking regions. The resulting plasmid was then transferred by conjugation into WSM1744 and double recombinants were selected as described previously [12].

For complementation of the WSM1744∆*sup3* mutant, a PCR-fragment encompassing the full *sup3*_*WSM1744*_ gene and the 500-bp upstream promoter region obtained with the high-fidelity enzyme Phusion DNA polymerase (Thermo Fischer Scientific) was cloned into the pVO155 vector [32] and re-introduced into WSM1744∆*sup3* by single-crossing over.

For introduction of *ernA*_*ORS3257*_ into USDA110 and USDA61 strains, PCR fragments corresponding to the promoter regions of *ernA*_*USDA110*_ or *ernA*_*USDA61*_ and the full *ernA*_*ORS3257*_ gene were merged by overlap extension PCR, subsequently cloned into the pVO155 vector [32], and introduced into USDA110 or USDA61 strains by single-crossing over.

For complementation experiments using the ORS3257∆*ernA* mutant, full-length *ernA*_*USDA110*_, *ernA*_*USDA61*_, *sup3*_*WSM1744*_, *sup3*_*NAS96-2*_ and *bel2-5*_*USDA61*_ were amplified as described above and cloned downstream of the *ernA*_*ORS3257*_ promoter into pVO155-pm-*ernA* (Supplementary Dataset S2) before re-introduction into ORS3257∆*ernA* by single-crossing over.

For site-directed mutagenesis of *ernA*, we used the plasmid pVO155-pm-*ernA::ernA*_*ORS3257*_ previously constructed to complement the ORS3257∆*ernA* mutant [12], the Q5 Site-Directed Mutagenesis Kit (NEB) and the primers indicated in Supplementary Dataset S2.

### Plant cultivation and symbiotic analysis

*A. indica* plants were cultured as previously described [5]. Eight plants per strain were inoculated and the symbiotic properties of the strain (number of nodules per plant, nitrogenase activity, infection of the nodules) were analyzed 21 days after inoculation as previously described [33]. The experiments were carried out at least in duplicate.

### Sup3 secretion analysis

*Sup3*_WSM1744_, *GFP* and *PopP2* coding sequences were introduced by LR recombination in pLAFR6-P2GFH/*hrpB* vector for expression of the protein of interest fused with a C-terminal 3x-HA epitope tag under the control of the HrpB-activated popP2 promoter sequence (Supplementary Dataset S2). The derived recombinant plasmids were introduced into *Ralstonia pseudosolanacearum* Δ*popP2* strain (GRS100 [34]) and Δ*popP2*/ Δ*hrcv* [35] strains by electroporation and selected on 5 µg/mL tetracycline and 7.5 µg/mL gentamicin. Production of *R. pseudosolanacearum* concentrated supernatants (from 10 mL of culture) was performed as previously described [36] with minor modifications. Briefly, transformed bacteria were grown in MP secretion medium (MP medium 1×, Congo Red 100 µg/mL, glutamate 20 mM and tetracycline 5 µg/mL) for 30 h at 28 °C under shaking. Pelleted cells from 1 mL of culture were lysed in 300 µL of 2× Laemmli buffer (pellet fraction). Proteins from the cell culture supernatant were precipitated with TCA 25% (v/v) for 12 h at 4 °C and resuspended in 50 µL of 2× Laemmli buffer. Protein extracts were denaturated for 5 min at 95 °C, loaded on 10% SDS-PAGE gels, and transferred on nitrocellulose membrane using Trans-Blot Turbo transfer kit (Bio-Rad) according to the manufacturer’s instructions. Blotted membranes were blocked with 5% milk in Tris-buffered saline plus 0.01% Tween 20 (TBS-T) for one hour at room temperature and subsequently incubated with desired antibodies at 4 °C overnight. Antibodies used were anti-HA-HRP (3F10, Roche, dilution 1:5000), polyclonal rabbit anti-GroEL (Stressgen Biotechnologies Corporation, dilution 1:20,000) and goat anti rabbit IgG-HRP (Bio-Rad, dilution 1:10,000). Chemiluminescent horseradish peroxidase (HRP) detection was performed using the Clarity Western ECL substrate (Bio-Rad).

### *Agrobacterium tumefaciens* infiltration assays in *Nicotiana benthamiana*

Binary plasmid *pB7FWG2-35S-eGFP-3Flag-sup3*_*WSM1744*_ used for constitutive expression of eGFP-3Flag-Sup3 (Supplementary Dataset S2), was introduced into *A. tumefaciens* strain GV3101 by electroporation [37]. Agro-infiltration of *N. benthamiana* leaves and localization of fluorescently labeled Sup3 were performed as previously described [12].

### Hairy root transformation with *Agrobacterium rhizogenes*

Plasmid *p35S-sup3* containing *sup3*_*NAS96-2*_ under the control of the 35S promoter (Supplementary Dataset S2), and the empty vector pJCV51 with the DsRed marker (https://gateway.psb.ugent.be), were introduced by electroporation into the *A. rhizogenes* Arqua1 strain used for hairy root transformations. *A. indica* root transformation was performed following previously described procedures [38].

## Results

### A large diversity of *Bradyrhizobium* strains nodulate *A. indica*

**Fig. 1:**
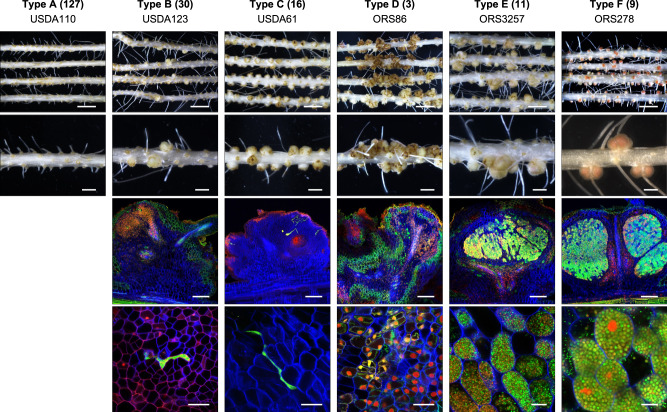
Symbiotic phenotypes of various *Bradyrhizobium* strains inoculated on *A. indica*. Six different symbiotic phenotypes were distinguished and designated as Type A to F (from the least to the most efficient symbiotic interaction) and for each phenotype a representative strain is shown. Type A: strains unable to nodulate. Type B: strains showing a weak nodulation (average < 5 nodules per plant) with sometimes intercellular infection. Type C: strains inducing a large number of nodules (average > 20 nodules per plant) with intercellular infection. Type D: strains forming massively multilobed nodules with intercellular infection. Type E: strains inducing misshapen and large nodules with a zone of intracellular infected plant cells but having a weak nitrogenase activity (no bacteroid differentiation). Type F: round-shaped nodules with a central zone containing intracellular infected plant cells with bacteria differentiated into bacteroids that efficiently fix nitrogen for the benefit of the plant. The number of strains in each category is shown in brackets. Row 1 and 2: photo of roots and nodules induced by the different strains. Scale bars: row 1, 0.5 cm; row 2, 0.2 cm. Row 3 and 4, Confocal microscopy images of section of nodules induced by the different strains observed after staining with SYTO 9 (green, live bacteria), propidium iodide (red, infected plant nuclei and dead bacteria or bacteria with compromised membranes) and calcofluor (blue, plant cell wall); Scale bars: row 3, 200 µm; row 4, 40 µm except for ORS3257 and ORS278 (10 µm).

We inoculated *A. indica* with 196 *Bradyrhizobium* strains and found that 69 strains induced nodules or nodule-like structures (Supplementary Dataset S1). Six distinct symbiotic phenotypes (named Type A to F) could be distinguished (Fig. 1). The Type A was the largest with 127 strains unable to induce nodules (Nod^-^). The 30 Type B strains showed a very low nodulation capacity, only a part of the inoculated plants displayed a low number (<5 per plant) of nodule-like structures and infection of these nodules was rare and limited to the intercellular spaces. The 16 Type C strains, including USDA61, induced a large number of nodules in all inoculated plants but the infection remained limited to the intercellular spaces as in Type B. Type D was observed for only three strains (BR2003, ORS86 and ORS111), and is characterized by a multilobed unorganized nodule development leading to numerous protuberances all along the root, which look more like galls than nodules. The presence of intercellular bacteria could sometimes be detected in these structures. In Type E (11 strains), which includes ORS3257, the elicited nodules were large, generally misshapen but with a clear intracellularly infected central tissue. However, as previously described [5], the intracellular bacteria did not differentiate properly into bacteroids. This is correlated with no or very low nitrogenase activity (Supplementary Dataset S1), which in all cases was insufficient to support plant growth (Supplementary Fig. S1). Finally, the Type F (9 strains), mainly represented by photosynthetic *Bradyrhizobium* strains, corresponds to bacteria inducing regular round-shaped nodules, infected intracellularly by bacteria differentiated into spherical bacteroids [39], which efficiently fixed nitrogen for the plant benefit. Thus, the ability to initiate nodule organogenesis in *A. indica* is widespread throughout the *Bradyrhizobium* genus but the outcome of this interaction strongly varies among the strains.

### Phylogenetic distribution of *nod*, *T3SS* and *ernA* genes in relation with the symbiotic properties

**Fig. 2:**
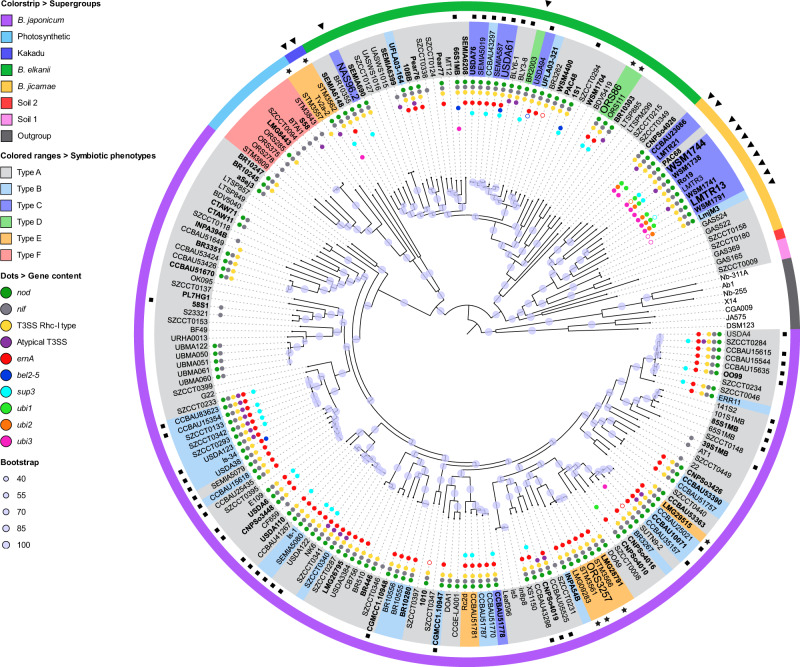
Maximum-likelihood phylogenomic tree of *Bradyrhizobium* strains tested in this study. The phylogeny was inferred from the core genome of the 196 *Bradyrhizobium* strains tested and several species from *Rhodopseudomonas* and *Nitrobacter* used as an outgroup. Purple circles on the nodes indicate ultrafast bootstrap values calculated by IQ-Tree. The presence of some symbiotic determinants in each strain is shown by colored dots referenced in the key, full dots indicate complete genes, empty dots indicate partial genes. Type strains are indicated in bold. All previously described supergroups [40] are present in our analyzed collection and indicated by a band strip color referenced in the key: *B. japonicum*, photosynthetic, Kakadu, *B. elkanii*, *B. jicamae*, Soil 1 and Soil 2. The symbiotic phenotype (Type A to F) of each strain is indicated by a colored range referenced in the key. The black triangles indicate strains inducing nodules while they lack *ernA*. The black stars indicate strains for which the genome has been sequenced in the frame of this study. The black squares indicate strains isolated from soybean nodule. The name of strains with a higher police size are those used for functional analyses in this study.

The assayed *Bradyrhizobium* strains cover the seven phylogenetic clades previously identified within the *Bradyrhizobium* genus and named *B. japonicum*, Photosynthetic, Kakadu, *B. elkanii*, *B. jicamae*, Soil 1 and Soil 2 Supergroups (Fig. 2) [40]. Correlating symbiotic properties with phylogenetic position showed that the Type F phenotype was restricted to the photosynthetic supergroup. Most of the strains of this group lack *nod* and *T3SS* genes. All other bacteria (59/69 strains) nodulating *A. indica* are scattered in 4 of the 6 other supergroups and all possess a classical T3SS of the Rhc-I type, except for the strain Tv2a-2 which has only an atypical T3SS [41]. Taken together, these observations suggest that the NF-independent, T3SS-independent symbiotic process is specific to strains belonging to the photosynthetic supergroup whereas the ability to use a T3SS-dependent process to induce nodulation is a more widespread trait found in a large variety of *Bradyrhizobium* strains.

We found that 45 of the 59 nodulating strains have an ErnA homolog (Supplementary Dataset S3), supporting the idea that ErnA is a widespread T3E in the *Bradyrhizobium* genus to trigger nodulation. However, correlation between the presence of ErnA and the ability to nodulate is not strict, as 33 strains having a complete ErnA homolog were Nod^-^ and on the contrary, 14 strains lacking an ErnA homolog nodulated *A. indica* (Fig. 2).

### Effect of ErnA variability on nodulation and infection

ErnA variants exist that differ by the presence or not of a 80 AA domain at its N-terminal end [12]. Furthermore, AA variations are also observed between ErnA orthologs. A phylogenetic tree of ErnA highlights its diversity (Supplementary Fig. S2A). In the branch that groups the highest number of ErnA proteins (34), a very high level of AA conservation is observed. Most of the strains of this phylogroup failed to nodulate (21 strains out of 34), while the remaining 13 showed a very weak nodulation ability (Type B phenotype), raising the question whether this form of ErnA is active. To analyze this, we introduced the *ernA* gene from the USDA110 strain (*ernA*_*USDA110*_) into the ORS3257∆*ernA* mutant which lost its nodulation ability [12]. The addition of the original *ernA* gene of ORS3257 restored nodulation while the introduction of *ernA*_USDA110_, put under the control of the *ernA*_ORS3257_ promoter, resulted only in weak nodulation (Supplementary Fig. S2B). This indicates that ErnA_USDA110_ is less active than ErnA_ORS3257_. Furthermore, when the *ernA* gene from ORS3257 was introduced into USDA110, the transformed strain (USDA110::*ernA*_ORS3257_) did not acquire nodulation ability (Supplementary Fig. S2D), indicating that factors other than ErnA are responsible for the Nod^-^ character of USDA110.

Important differences in the nodule infection were also observed between strains using a T3SS-dependent process, with some inducing only intercellularly infected nodules (Type B, C or D) and others inducing intracellularly infected nodules (Type E). We investigated whether these differences could be related to variations in the ErnA sequence, and thus whether ErnA could control both nodulation and infection as NFs do. To address this question, we introduced the *ernA* gene from USDA61 (*ernA*_USDA61_) (Type C) into the ∆*ernA* mutant of ORS3257 (Type E). No difference in nodule number and the type of infection was observed between plants inoculated with WT ORS3257 and the ORS3257∆*ernA::ernA*_*USDA61*_ mutant (Supplementary Fig. S2B, C). Similarly, introduction of *ernA*_ORS3257_ into USDA61 did not promote intracellular infection (Supplementary Fig. S2E). This data suggests that the main function of ErnA is dedicated to nodule organogenesis but not infection.

### Strain NAS96.2 contains a novel putative ET-Nod

**Fig. 3:**
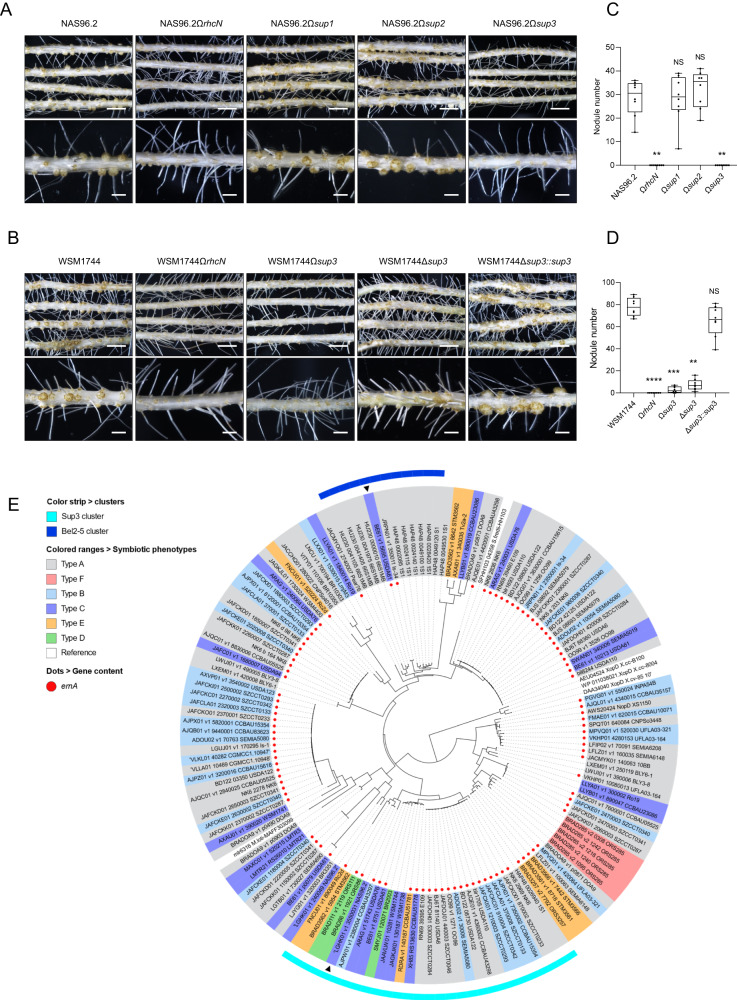
Sup3, a putative SUMO-protease conferring the ability to nodulate *A. indica*. Images of roots and nodules of *A. indica* plants at 21 days post inoculation with strain NAS96.2 and its mutant derivatives in T3SS (Ω*rhcN*) and in different genes encoding putative SUMO-proteases (Ω*sup1*, Ω*sup2* and Ω*sup3*) (**A**) and strain WSM1744 containing a *sup3* homolog and its mutant derivatives (Ω*rhcN*, Ω*sup3*, ∆*sup3* and ∆*sup3::sup3)* (**B**) (scale bars: upper panels 0.5 cm, lower panels 0.2 cm). Nodule number of *A. indica* plants at 21 days after inoculation with NAS96.2 (**C**) or WSM1744 (**D**) and their respective mutants. Box plots show the results of one of the two experiments performed independently (8 plants each). **p ≤ 0.001, significant differences between the wild-type strain NAS96.2 and each mutant (**C**) and **p ≤ 0.005, ***p < 0.0005, ****p < 0.0001, significant differences between the wild-type strain WSM1744 and each mutant (**D**) using a nonparametric Kruskal–Wallis test, NS not significant. **E** Phylogenetic tree of full-length SUMO-proteases identified in the collection of *Bradyrhizobium* strains used in this study. Considering the very different branch lengths, the MAD (minimal ancestor deviation) method was used for rooting the tree [59]. The SUMO-proteases Sup3 from NAS96.2 and Bel2-5 from USDA61 (indicated by black triangles) belong to two distinct phylogroups (indicated by a color strip). The symbiotic phenotype of the strains in which the SUMO-protease are identified is indicated by a colored range referenced in the key and the presence of an *ernA* homolog by a red circle. As references (white range), the following SUMO-proteases are included: (i) mlr6316 identified in *Mesorhizobium loti* MAFF303099 [60]; (ii) SFHH103_04358 identified in *Sinorhizobium fredii* HH103 [61] and (iii) The XopD SUMO-proteases [57] identified in various *Xanthomonas campestris* strains.

As mentioned above, 14 strains lacking an *ernA* homolog can nodulate *A. indica* (Fig. 2). This raises the question: Do these strains use a T3SS-independent mechanism or an alternative T3E that mimics the action of ErnA? We focused on the NAS96.2 strain, which is closely related to three strains (SEMIA6148, SEMIA690, BR1035) unable to nodulate *A. indica* but having a T3SS (Fig. 2), suggesting the possibility that NAS96.2 has a specific ET-Nod that is absent in these three related strains. By comparing their putative effectome (Supplementary Dataset S4), we identified three strain-specific T3Es in NAS96.2, that we annotated Sup1, Sup2 and Sup3, considering that all of them encode putative SUMO-proteases. Insertional mutants of these three genes were constructed as well as a T3SS mutant (insertion mutant in the *rhcN* gene). The *rhcN* mutation abolished nodulation, confirming that the process is dependent on T3SS (Fig. 3A, C). Furthermore, while the *sup1* and *sup2* mutants behave like the WT strain, the *sup3* mutant did not induce nodules (Fig. 3A, C), suggesting that in the NAS96.2 strain, Sup3 is an ET-Nod controlling nodulation of *A. indica*.

### A sub-class of SUMO-proteases in *Bradyrhizobium* genus confers nodulation ability

The *sup3* gene (LGHK01_v1_3550031) encodes a very large protein of 1463 AA which displays some similarities with Bel2-5 and carries at its C-terminus, a SUMO-protease domain of the C48 peptidase [ubiquitin-like protease 1 (ULP1)] family. Considering that SUMO-proteases were previously identified as one of the largest classes of T3Es in bradyrhizobia [41] and that two of them (Bel2-5 and Sup3) could act as ET-Nod, we examined in more details the distribution of SUMO-proteases in our strain collection. By searching for the presence of the ULP1 domain in the proteomes of these bacteria, we identified a total of 228 SUMO-proteases present in 91 strains, all of them containing a T3SS reinforcing the idea that these SUMO-proteases correspond to T3Es (Supplementary Dataset S5).

A phylogenetic analysis on the 149 available full-length SUMO-protease sequences (Supplementary Dataset S5) revealed that Bel2-5 and Sup3 belong to two distinct clades (Fig. 3E). Plotting on this tree the symbiotic properties of the corresponding strains reveals that strains of the Sup3 cluster predominantly nodulated *A. indica*. Furthermore, among these strains, four of them, in addition to NAS96.2, lack *ernA*, suggesting that the presence of a Sup3-like SUMO-protease might confer the ability to nodulate *A. indica*. To support this hypothesis, we constructed *sup3* (Ω*sup3)* and T3SS (Ω*rhcN)* insertional mutants in the *B. archetypum* WSM1744 strain. The T3SS mutation completely aborted the nodulation and the *sup3* mutation also resulted in a drastic reduction of nodules (Fig. 3B, D). Furthermore, the ∆*sup3* deletion mutant had the same phenotype as the Ω*sup3* insertional mutant and re-introduction of *sup3*_WSM1744_ restored nodulation to WSM1744 wild-type level (Fig. 3B, D) confirming that the nodulation deficiency of *sup3* mutants was specifically due to *sup3* inactivation. Taken together, these data indicate that the nodulation between WSM1744 and *A. indica* is T3SS-dependent and that Sup3, as in NAS96.2, is the main T3E governing nodule organogenesis.

### Several *Bradyrhizobium* strains contain multiple putative ET-Nods

**Fig. 4:**
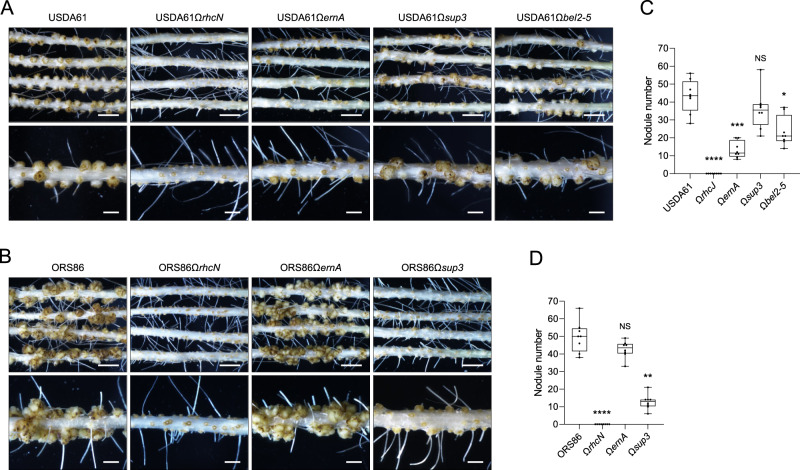
The ET-Nod governing nodulation differs according to the strain. Images of roots and nodules of *A. indica* plants at 21 days post inoculation with strain USDA61 (**A**) or ORS86 (**B**) and their respective mutants in T3SS (Ω*rhcJ* or Ω*rhcN*) and several identified ET-Nods (Ω*ernA*, Ω*sup3*, Ω*bel2-5*) (scale bars: upper 0.5 cm, lower 0.2 cm). Nodule number on *A. indica* plants at 21 days after inoculation with USDA61 (**C**) or ORS86 (**D**) and their respective mutants. Box plots show the results of one of the two experiments performed independently (8 plants each). *p < 0.05, **p < 0.005, ***p ≤ 0.0005, ****p < 0.0001, significant differences between the wild-type strain and each mutant using a nonparametric Kruskal–Wallis test, NS not significant. The mutants USDA61Ω*ernA*, USDA61Ω*rhcJ* and USDA61Ω*bel2-5* were previously obtained [12, 62, 63].

Among the group of strains possessing a *sup3* homolog, we observed that some have additional putative ET-Nods. This is the case of ORS86 which carries a *sup3* and *ernA* homolog, and USDA61 which contains in addition to *sup3* and *ernA* also *bel2-5* (Fig. 2). This raises the question whether these different ET-Nods have cumulative effect in nodule induction, especially in the case of ORS86 which induced abundant nodulation in *A. indica* (Fig. 1). Analysis of the symbiotic properties of the ORS86 and USDA61 mutants in these different ET-Nods and in the *rhcN* gene (Fig. 4), showed that i) the abundant nodulation of ORS86 was T3SS-dependent, as observed in USDA61 (Fig. 4A, B), and ii) only one of the putative ET-Nods plays a prominent role in nodulation, Sup3 in the ORS86 strain and ErnA in USDA61 (Fig. 4C, D).

During the interaction between USDA61 and *Glycine max* cv Enrei, it was shown that Bel2-5 controls nodulation, whereas the ErnA homolog plays only a minor role [13]. Thus, the ET-Nod governing the nodulation process is strain-dependent but also host plant-dependent.

### Introduction of *sup3*, but not *bel2-5*, restores nodulation by ORS3257∆*ernA*

**Fig. 5:**
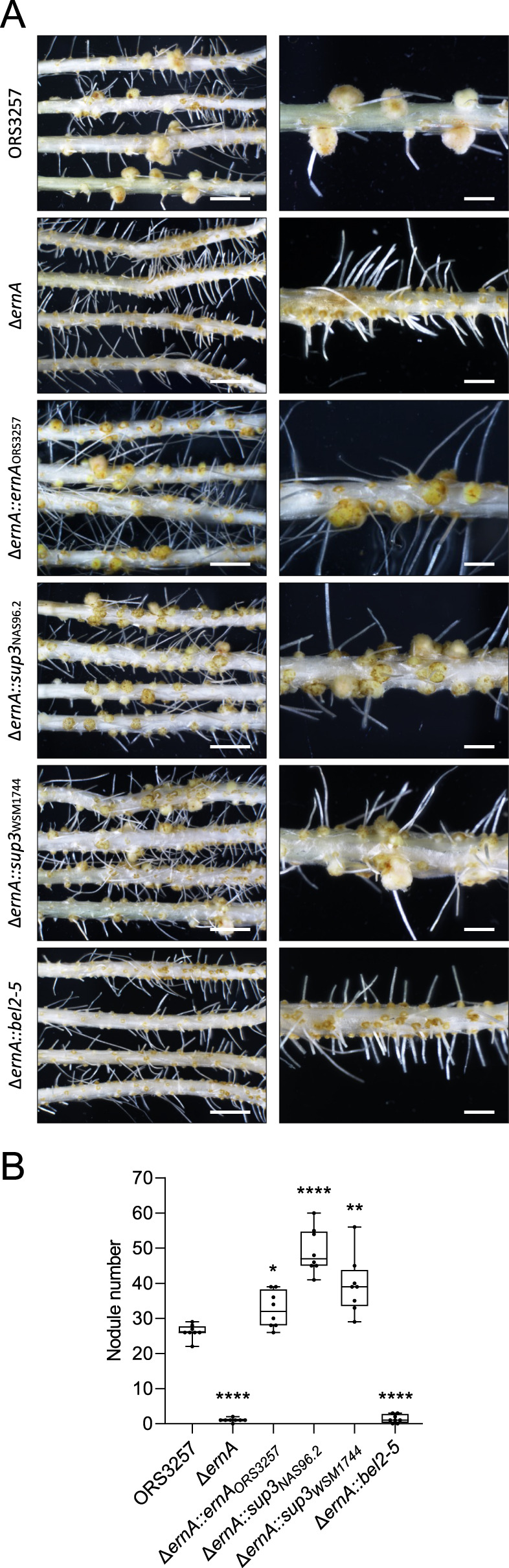
*sup3*, but not *bel2-5*, complements nodulation of ORS3257∆*ernA* mutant. **A** Symbiotic phenotypes of ORS3257 and various derivatives of the ORS3257∆*ernA* mutant carrying *ernA*, *sup3* and *bel2-5* genes (∆*ernA::ernA*_ORS3257_; ∆*ernA::sup3*_NAS96.2_; ORS3257∆*ernA::sup3*_WSM1744_; ORS3257∆*ernA::bel2-5*) (scale bars: left 0.5 cm, right 0.2 cm). **B** Nodule number on *A. indica* plants at 21 days after inoculation with ORS3257 and the ORS3257∆*ernA* mutant derivatives. Box plots show the results of one of the two experiments performed independently (8 plants each). *p ≤ 0.01, **p < 0.005, ****p < 0.0001, significant differences between the wild-type strain and each mutant using a parametric Welch test.

To further demonstrate that Sup3 acts as an ET-Nod, we used a gain of function approach by analyzing the ability of Sup3 to complement the nodulation-deficient ORS3257∆*ernA* mutant. For this purpose, the *sup3* gene from WSM1744 and NAS96.2 under the control of the *ernA* promoter was introduced into ORS3257∆*ernA*. We also tested the complementation with the *bel2-5* gene from USDA61. Introduction of *sup3*_*WSM1744*_ or *sup3*_*NAS96.2*_ but not of *bel2-5* restored the ability of the mutant to induce intracellularly infected nodules on *A. indica * (Fig. 5). In both cases the number of formed nodules was greater than obtained with WT ORS3257 (Fig. 5A, B). This data confirms the ability of Sup3 to trigger nodulation on *A. indica*, which in the ORS3257 background, seems to be higher than ErnA.

### The ectopic expression of *sup3* in *Aeschynomene* spp. roots induces spontaneous nodules

**Fig. 6:**
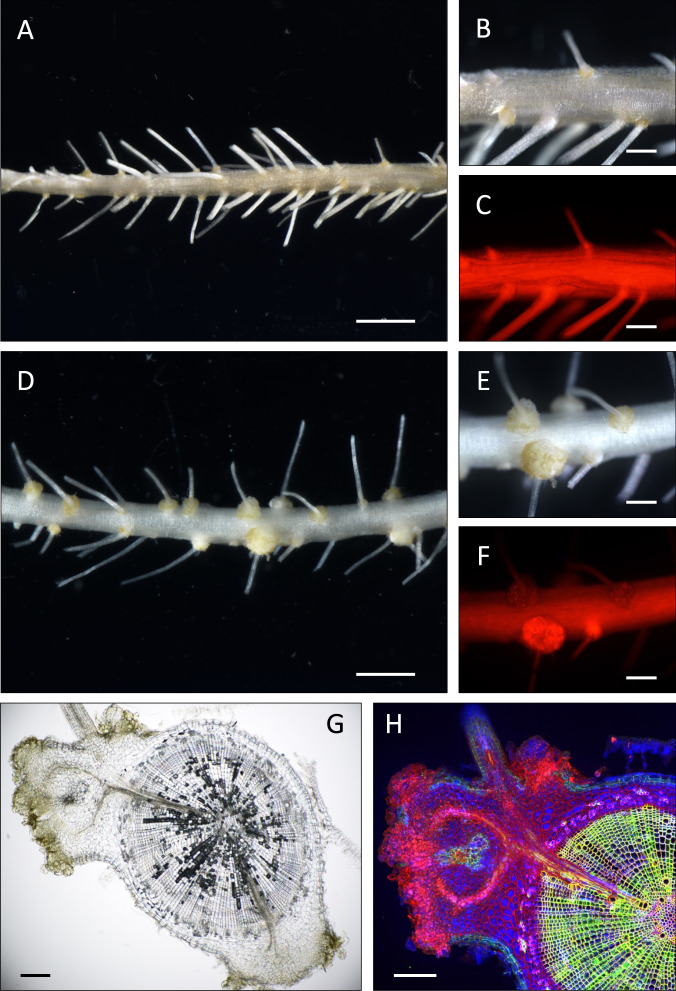
Ectopic expression of *sup3* in *A. evenia* roots induces spontaneous nodules. *A. evenia* roots transformed either with the empty vector containing the DsRed marker (**A**–**C**) or with *p35S-sup3* (**D**–**F**) at 7 weeks after transformation (no bacterial inoculation). Roots were observed by a fluorescence stereomicroscope equipped with a DsRed filter. Scale bars: **A** and **D**, 2 mm; **B**, **C**, **E** and **F**, 500 µm. Micro-sections of pseudo-nodules observed using light microscopy showing the peripheral vascularization (**G**) and using a confocal microscope (**H**) after staining with SYTO 9, propidium iodide and calcofluor showing that the induced pseudo-nodules do not contain bacteria. Scale bars **G** and **H**, 200 µm.

To analyze if Sup3 alone can induce nodule organogenesis, transgenic lines overexpressing *sup3*_NAS96.2_ were generated in two *Aeschynomene* species (*A. indica* and *Aeschynomene evenia)*. Seven weeks after *Agrobacterium rhizogenes* transformation, about 30% and 40% of the transformed roots of *A. indica* and *A. evenia*, respectively, contained nodule-like structures with an average of 5 to 10 nodules per nodulated plant (Fig. 6D–F and Supplementary Fig. S3). These spontaneous nodules were observed at the level of the emergence of lateral roots and contained peripheral vascular bundles, similar to nodules induced by the bacterium (Fig. 6G, H and Supplementary Fig. S3). These nodule-like structures were not observed on roots transformed with the empty vector (Fig. 6A–C and Supplementary Fig. S3). Thus, ectopic expression of *sup3* is sufficient to activate the nodule organogenesis program in *Aeschynomene*.

### Sup3 is a bona fide T3E that targets the host nucleus

**Fig. 7:**
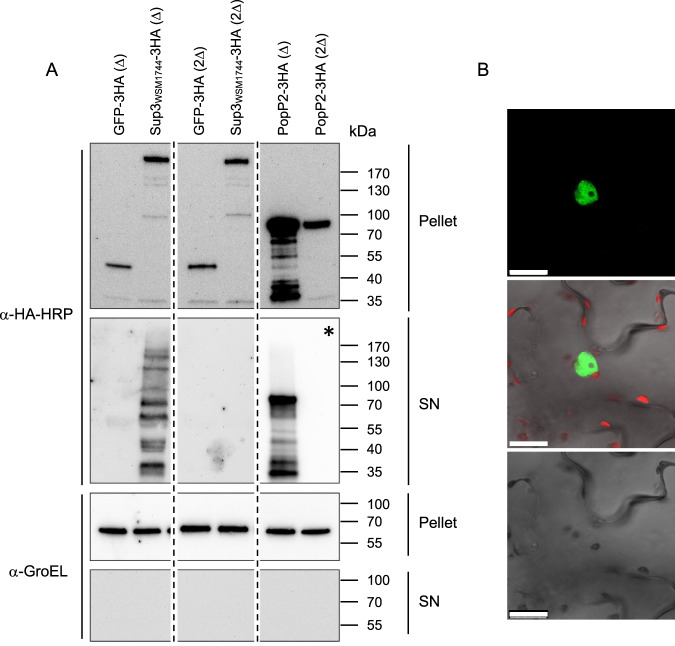
Sup3_WSM1744_ is secreted through the T3SS machinery of *Ralstonia pseudosolanacearum* and is targeted to the plant nucleus. **A** GFP-3HA, Sup3_WSM1744_-3HA and PopP2-3HA either produced in Δ*popP2 (∆)* or Δ*popP2*/Δ*hrcV (2∆)* bacterial cells were immuno-detected from pellet and supernatant fractions using an anti-HA antibody. Like PopP2, Sup3_WSM1744_ could not be detected in culture supernatants of *ΔpopP2/ΔhrcV* bacteria cells, demonstrating its proper secretion by the T3SS. All protein samples were probed with anti-GroEL antibody raised against the cytoplasmic chaperonin GroEL to ensure that no bacterial lysis had occurred during the preparation of supernatant fractions. The star (*) indicates a shorter exposure of the membrane with lanes corresponding to the supernatants of Δ*popP2* and Δ*popP2*Δ*hrcv* strains expressing PopP2-3HA. Dotted lines indicate unnecessary lanes that have been removed from the same membranes. **B** GFP fluorescence observed in *N. benthamiana* leaf cells transiently expressing eGFP-3Flag-Sup3_WSM1744_. GFP fluorescence signal was visualized by confocal microscopy 48 h after *Agrobacterium* infiltration. From top to bottom: the GFP fluorescence spectrum of a representative nucleus from a transformed cell, an overlay of GFP and chlorophyll fluorescence and a bright field view of the scanned cell (Scale bars: 25 μm).

To check whether Sup3 can be secreted through the T3SS machinery, we introduced a C-ter His_6_-tagged version of Sup3 into WSM1744 and its ∆*T3SS* mutant. However, despite several attempts, we were unable to detect Sup3-His_6_ in both culture supernatant and cell pellets suggesting that synthesis of the tagged protein is too low to be detected. The extremely slow growth of the WSM1744 strain and the very large size of the Sup3 protein could be the reason for the difficulties in detection. Therefore, we used as an alternative approach a Type 3 secretion assay originally developed in the pathogenic bacterium *Ralstonia pseudosolanacearum*. Here, Sup3_WSM1744_ was fused with a C-terminal 3x-HA epitope tag and expressed in a Δ*popP2* strain that has been previously used to demonstrate the T3SS-dependent secretion of the *R. pseudosolanacearum* T3E PopP2 [36]. GFP and PopP2 were used as negative and positive controls, respectively. As expected, Sup3_WSM1744_ was detected in the pellet and the supernatant fractions, indicating that, like PopP2, Sup3_WSM1744_ can be secreted through the T3SS (Fig. 7A). Of note, Sup3_WSM1744_ and PopP2 were strongly processed after secretion by a mechanism that remains to be determined. By contrast, GFP that lacks a secretion signal was only detected in the pellet fraction. When expressed in a Δ*popP2*/*ΔhrcV* strain mutated both in *popP2* and in *hrcV* that encodes a conserved inner membrane component of the T3SS [42], Sup3_WSM1744_ and PopP2 were absent in the culture supernatant confirming that their secretion was T3SS-dependent.

To investigate its subcellular localization *in planta*, Sup3_WSM1744_ N-terminally fused with enhanced GFP and a triple hemagglutinin epitope tag (eGFP-3Flag-Sup3_WSM1744_) was transiently expressed in tobacco leaf cells. As previously observed with ErnA [12], eGFP-3Flag-Sup3 was found to be targeted to the plant nucleus, suggesting that Sup3 triggers nodule organogenesis by interfering with host nuclear components (Fig. 7B).

### Identification of three putative ET-Nods, Ubi1, Ubi2 and Ubi3, in *Bradyrhizobium icense* LMTR13

**Fig. 8:**
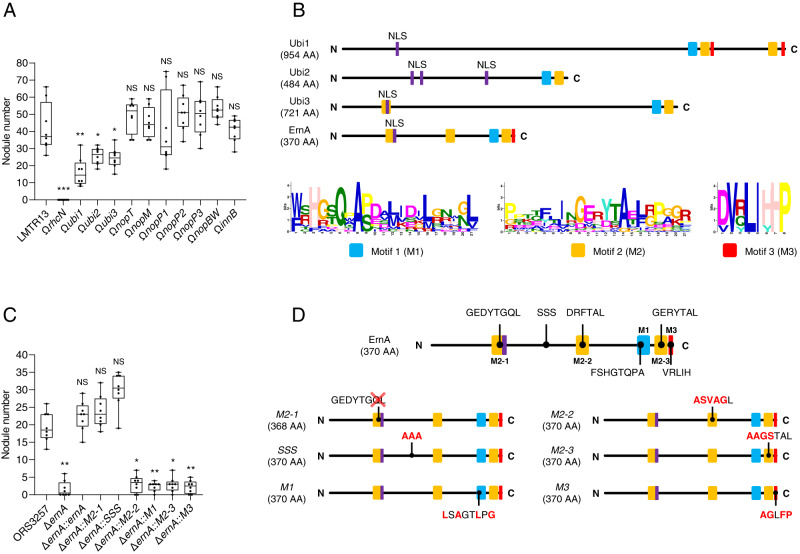
The putative T3Es (Ubi1, Ubi2 and Ubi3) from *B. icense* LMTR13 share conserved motifs with ErnA that are important for nodulation in *A. indica*. **A** Nodule number on *A. indica* plants at 21 days after inoculation with strain LMTR13 and its mutant derivatives in T3SS (Ω*rhcN*) and various putative T3Es. Box plots show the results of one of the two experiments performed independently (8 plants each). *p < 0.05, **p < 0.005, ***p < 0.0005 significant differences between the wild-type strain and each mutant using a nonparametric Kruskal-Wallis test, NS: not significant. **B** Schematic representation of ErnA and the 3 new putative T3Es (Ubi1, Ubi2 and Ubi3). The conserved motifs (M1, M2 and M3) identified by MEME analysis (Fig. S4) are shown by colored squares and in web logos. The putative NLS (Nuclear localization signal) predicted by Localizer [43] are indicated by purple lines. **C** Nodule number on *A. indica* plants at 21 days after inoculation with ORS3257 and the ORS3257Δ*ernA* mutant expressing ErnA with mutations in the different identified motifs indicated in panel B. In the M2-1 motif, the mutagenesis was focused on the QL residues (deletion) considering that these residues are absent in the ErnA sequences clustering with ErnA_USDA110_ shown to be less active in nodulation triggering on *A. indica* (Supplementary Fig. S2). Box plots show the results of one of the two experiments performed independently (8 plants each). *p ≤ 0.01, **p < 0.005, significant differences between the wild-type strain and each mutant using a nonparametric Kruskal-Wallis test, NS: not significant. **D** Schematic representation of the different tested mutated forms of ErnA. The indicated SSS motif was identified as a putative DNA binding site using the software program DP Bind [45].

Except for the PAC68 strain, all ten tested strains from the *B. jicamae* supergroup nodulated *A. indica* while all lack an *ernA* homolog and most also lack a *sup3* homolog, suggesting the existence of other ET-Nod(s) within the strains of this phylogroup (Fig. 2). Comparative analysis of the effectome of these different strains identified only one common T3E (NopM) present specifically in nodulating strains (Supplementary Dataset S4). However, this T3E, which is among the most commonly found and studied T3Es in rhizobia [41], has never been reported to have a nodulation induction activity. To identify ET-Nods in these strains, we focused on the *B. icense* LMTR13 strain, which has the smallest effectome among the considered nodulating strains. We mutated the ten *T3E* genes, which are present in this strain but absent in PAC68 as well as the T3SS apparatus (*rhcN* mutant). Once again, we confirmed the critical role played by T3SS in this symbiotic process with the absence of nodulation observed for the *rhcN* mutant (Fig. 8A). Among the ten *T3E* mutants tested, 3 mutants in previously unknown *T3E* genes, which we named Ubi1, Ubi2 and Ubi3 (Ubi for Unknown T3Es identified in the *Bradyrhizobium icense* strain), gave a significant reduction in nodule number of 62, 40 and 42%, respectively, compared to the WT strain. The absence of a mutant with (nearly) completely abolished nodulation capacity, as observed for the *ernA* or *sup3* mutants in other strains, leads us to hypothesize that these three putative T3Es act synergistically.

Ubi1, Ubi2 and Ubi3 show no homology to known functional domains, but a nuclear localization signal can be predicted for each of them using Localizer [43] suggesting that these T3Es are also targeted to the host nucleus (Fig. 8B). Their distribution is relatively restricted; Ubi1, Ubi2 and Ubi3 homologs can be detected respectively in 8, 7 and 13 strains of the bradyrhizobia collection used here, among which a large proportion of strains belong to the *B. jicamae* supergroup (Fig. 2 and Supplementary Dataset S3).

### Ubi1, Ubi2 and Ubi3 share common conserved motifs found in the C-terminus of ErnA

As the three new putative ET-Nods are proteins with unknown function, we used the MEME suite tools [44] to investigate if Ubi1, Ubi2 and Ubi3 share common motifs with other known and characterized T3Es. This analysis revealed that these three T3Es share two contiguous 21 AA motifs (M1 and M2), which are also found at the C-terminal end of ErnA (Fig. 8B and Supplementary Fig. S4). Furthermore, Ubi1 and ErnA share an additional 8 AA motif (M3) downstream of M1 and M2. This suggests a possible functional link between these different T3Es. The succession of M1 and M2, associated or not with M3, is also found in other T3Es (NopAR, InnB, NopL and NopAB) (Supplementary Fig. S4). The latter two were previously shown to play a role in the T3SS-dependent symbiosis [12, 13].

The bioinformatic tool DP-bind [45] recognized the M2 motif as a DNA-binding site while the GPS-SUMO tool [46] predicted that the M3 domain is a SUMO interaction motif (SIM), a motif found in non-covalently SUMO-binding proteins [47]. Note that the M2 motif is found three times in ErnA (Fig. 8B). To gain further insight into the importance of these conserved motifs, we constructed different mutated forms of ErnA removing or modifying these motifs and tested their ability to complement the ORS3257∆*ernA* mutant (Fig. 8C, D). We also included a mutagenesis of the SSS motif, which was also predicted as a putative DNA-binding site by DP-Bind. While ErnA^M2-1^ and ErnA^SSS->AAA^ restored nodulation ability of the ORS3257∆*ernA* mutant, the ErnA variants mutated in the M1, M2-(2/3) and M3 motifs failed to do so (Fig. 8C, D). These data highlight the importance of these different motifs in ErnA functionality.

## Discussion

### The NF-independent T3SS-independent symbiosis, a process restricted to the photosynthetic *Bradyrhizobium* supergroup

Using a large number of *Bradyrhizobium* strains, this study shows that the ability to nodulate *A. indica* via a NF-independent, T3SS-independent process is restricted to *Bradyrhizobium* strains belonging to the photosynthetic supergroup. The name of this group is misleading because it includes the non-photosynthetic strain STM3843, which also nodulates *A. indica* [48], showing that the photosynthetic character is not a determining factor in this process. In agreement, an ORS278 mutant lacking the photosystem reaction center (∆*pufLM*) nodulates *Aeschynomene sensitiva* roots as efficiently as the wild-type strain [49]. However, bacterial photosynthesis, gives these bacteria a functional advantage in stem nodulation, another rare property found in some *Aeschynomene* species, likely by contributing to the energetic requirement of nitrogenase and to the survival of the bacteria on the stem surface [49]. The molecular basis of this symbiotic process is still unknown but the recent identification of plant determinants involved in this symbiosis, with in particular the identification of a new putative membrane receptor [50], should allow to progress in the identification of the bacterial signal governing this symbiosis.

### The NF-independent T3SS-dependent symbiosis, a more common process

Outside the photosynthetic supergroup of bradyrhizobia, all 59 strains inducing nodules or nodule-like structures on *A. indica* have in common that they harbor a *T3SS* gene cluster. Inactivating a key component (RhcN) in the T3SS machinery in representative strains resulted in all cases in a loss of nodulation confirming the key role of T3SS. More than 50% of the tested strains that have a T3SS are able to initiate a symbiosis. Nevertheless, none of them is capable of efficiently fixing nitrogen with *A. indica* (Supplementary Fig. S1). This does not seem to be correlated with the absence of the *nifHDK* genes, encoding the protein subunits of the nitrogenase enzyme complex, since these genes are present in all these strains, except for STM3557 (Fig. 2), but rather with an inability to differentiate properly into bacteroids, which corresponds to the active form of rhizobia capable of fixing nitrogen [39]. This raises the questions what is missing in these strains to be fully functional and if in nature a symbiosis solely dependent on a T3SS really exists? Indeed, it is intriguing that, except the Tv2a-2 strain, all the strains able to develop a T3SS-dependent symbiosis with *A. indica* also contain *nod* genes. These genes are dispensable in the context of *A. indica* but are necessary in almost all other *Bradyrhizobium*-legume interactions. So, what is the advantage for these bacteria to have both NFs and ET-Nods? Do these ET-Nods act synergistically with NFs to reinforce the nodulation efficiency of the strains on their natural NF-dependent host plants? Can the ET-Nods substitute for the NFs in case they are not properly recognized by the LysM-RLK plant receptors to enlarge the host spectrum of the bacteria?

*Bradyrhizobium* strains isolated from soybean nodules represent a high proportion of sequenced strains given the agronomic importance of this plant. Three-quarter of them (33/44) contain an ErnA homolog, while the other ET-Nods (Bel2-5, Sup3, Ubi1, Ub2 and Ubi3) are much less frequent (Fig. 2). Despite the high diversity of these soybean-nodulating strains, their ErnA proteins are strongly conserved (Supplementary Fig. S2). Although this ErnA variant seems less active in triggering nodulation in *A. indica* (Supplementary Fig. S2), we can assume that a strong selective pressure must be exerted on this ET-Nod during the *Bradyrhizobium-Glycine max* interaction to maintain such a high degree of conservation. ErnA and possible also other ET-Nods could therefore indeed play an important role in the fine tuning of the interaction between bradyrhizobia and their host plants, even if these strains dispose NFs to trigger nodulation.

### Distinct ET-Nods induce nodule organogenesis

ErnA in strain ORS3257 is the first identified ET-Nod shown to activate the nodulation process in *A. indica* [12]. This T3E is conserved in more than two thirds of *Bradyrhizobium* strains with a T3SS (Fig. 2). The widespread ability among *Bradyrhizobium* strains to develop T3SS-dependent nodulation is most likely related to this high degree of conservation of this effector in strains having a T3SS. The loss of nodulation capacity observed with *ernA* mutants of various strains (ORS3257, USDA61) and the gain of nodulation observed after transferring ErnA in strain lacking it (DOA9) [12], support this hypothesis. However, this link between nodulation capacity and the presence of *ernA* is not strict as our study shows multiple examples of non-nodulating strains that harbor *ernA* and of strains that nodulate but do not contain *ernA*. Several non-exclusive reasons may explain the inability of some strains to nodulate *A. indica* while containing *ernA*: i) possessing a variant form of ErnA that would be less or not functional in the *A. indica* context, ii) the co-secretion of other T3Es triggering immune responses or iii) the presence or absence of other determinants involved in the recognition of the bacterium by the plant, such as EPS, KPS or LPS, known to modulate symbiotic and immune responses [51–53].

Among the strains able to nodulate in the absence of ErnA, we identified several new ET-Nod genes able to mimic the action of *ernA*. The first one, *sup3*, identified in several strains (NAS96.2, WSM1744 and ORS86) codes for a putative SUMO-protease. SUMO is a small protein related to the ubiquitin family that is used by eukaryotic cells to modify post-transcriptionally a large diversity of proteins [54]. The addition of the SUMO (SUMOylation process) or its cleavage by SUMO-proteases (deSUMOylation process) can completely modify the biology of the targeted proteins, such as their localization, their interaction with other partner proteins, their enzymatic activity and their stability [55, 56]. SUMO-proteases are one of the most abundant classes of T3Es identified in bradyrhizobia [41]. Most of these SUMO-proteases, including Sup3 and Bel2-5, display the same architecture [56]. They correspond generally to very large proteins, often above one thousand AA, with a conserved C-terminal region corresponding to the SUMO-protease catalytic domain and a large N-terminal region containing no known functional domain. Phylogenetic analysis showed that the strains which have a SUMO-protease phylogenetically close to Sup3 in general nodulate *A. indica* and we confirmed in three of them (NAS96.2, WSM1744 and ORS86), the key role of Sup3 in controlling the nodulation process. In contrast, the SUMO-protease Bel2-5, which was found to be the key T3E regulating nodulation during the USDA61-*Glycine max* interaction, does not appear to play an important role in the context of *A. indica*. This indicates that there is a host plant-dependent specificity of nodulation activation of these SUMO proteases that is most likely related to their N-terminal region that is highly variable and supposed to be involved in substrate specificity [56]. Given the large diversity of SUMO-proteases present in bradyrhizobia, we expect that among them there are other ET-Nods able to activate nodulation on other host legumes.

How the different identified SUMO-proteases in *Bradyrhizobium* strains act to trigger the nodulation process remains unknown. A similar mechanism as used by the T3E XopD, present in some pathogenic bacteria of the genus *Xanthomonas*, which acts by deSUMOylation of a specific transcription factor (TF), can be assumed [57]. However, to our knowledge, none of the identified actors in legumes known to be recruited to the symbiotic signaling pathway [2, 50] have been reported to date to be modified by SUMOylation. To understand their mechanism in nodulation, future research should thus focus on the identification of the target(s) of these ET-Nod SUMO-proteases.

We observed that the addition of *sup3* to an *ernA* mutant can restore nodulation. This result is counterintuitive given that these two proteins are completely distinct. Two hypotheses can explain this result: i) these two ET-Nods trigger the same nodulation signaling pathway but acting on distinct actors at different levels of this pathway or, ii) these two ET-Nods could manipulate the same component(s) of this pathway but in different ways. Considering that the other putative ET-Nods (Ubi1, Ubi2 and Ubi3) identified in *B. jicamae* supergroup strains share certain motifs found in ErnA, this suggests a possible functional link between these different ET-Nods. In particular, a putative SIM domain was identified in Ubi1 and ErnA that appears to be essential for the nodulation induction activity of ErnA. This type of domain is found in many eukaryotic proteins that interact non-covalently with SUMO [47, 58]. All this would support the hypothesis that several identified ET-Nods have the same common SUMOylated target(s) but interact in a different way. For Sup3 and Bel2-5, this interaction would result in deSUMOylation of the target(s) and for ErnA and Ubi1 a modification of activity of the target(s) via a non-covalent protein-protein interaction thanks to the SUMO and the SIM domains. As the interaction of ErnA with SUMOs has to be validated and further functional studies are necessary to confirm that Ubi1, Ubi2 and Ubi3 are bona fide ET-Nods, this hypothesis remains speculative. However, this gives us some avenues to explore in future research to advance in the general understanding of the *modus operandi* of this newly discovered class of T3Es triggering nodulation.

In conclusion, this study provides much information on the factors predisposing *Bradyrhizobium* strains to develop a non-canonical symbiosis with legumes that is not based on NF perception. Many gaps remain, including the identification of the signals used by photosynthetic *Bradyrhizobium* strains to trigger nodulation and the understanding of the mode of action of the identified ET-Nods. It is important to continue this research on these alternative symbiotic systems to better estimate their occurrence and importance to trigger nodulation in nature, both in wild and crop legumes. Their understanding can shed new light on the evolution of symbiotic nitrogen-fixing mechanisms but also provide new levers to improve legume crop yields by optimizing their symbiotic capacity.

## Supplementary information

Figure S1

Figure S2

Figure S3

Figure S4

Dataset 1

Dataset 2

Dataset 3

Dataset 4

Dataset 5

## Data Availability

The genomic sequences of the 8 *Bradyrhizobium* strains sequenced in this study are available at NCBI BioProject (accession: PRJNA944954).

## References

[1] Oldroyd GED (2013). Speak, friend, and enter: signalling systems that promote beneficial symbiotic associations in plants. Nat Rev Microbiol.

[2] Roy S, Liu W, Nandety RS, Crook A, Mysore KS, Pislariu CI (2020). Celebrating 20 years of genetic discoveries in legume nodulation and symbiotic nitrogen fixation. Plant Cell.

[3] Giraud E, Moulin L, Vallenet D, Barbe V, Cytryn E, Avarre JC (2007). Legumes symbioses: absence of *nod* genes in photosynthetic bradyrhizobia. Science..

[4] Okazaki S, Kaneko T, Sato S, Saeki K (2013). Hijacking of leguminous nodulation signaling by the rhizobial type III secretion system. Proc Natl Acad Sci USA.

[5] Okazaki S, TIttabutr P, Teulet A, Thouin J, Fardoux J, Chaintreuil C (2016). Rhizobium–legume symbiosis in the absence of Nod factors: two possible scenarios with or without the T3SS. ISME J.

[6] Mornico D, Miche L, Bena G, Nouwen N, Vermeglio A, Vallenet D (2012). Comparative genomics of *Aeschynomene* symbionts: insights into the ecological lifestyle of *nod*-independent photosynthetic bradyrhizobia. Genes.

[7] Notti RQ, Stebbins CE (2016). The structure and function of Type III secretion systems. Microbiol Spectr.

[8] Hajra D, Nair AV, Chakravortty D (2021). An elegant nano-injection machinery for sabotaging the host: Role of Type III secretion system in virulence of different human and animal pathogenic bacteria. Phys Life Rev.

[9] Marie C, Broughton WJ, Deakin WJ (2001). Rhizobium type III secretion systems: legume charmers or alarmers?. Curr Opin Plant Biol.

[10] Staehelin C, Krishnan HB (2015). Nodulation outer proteins: double-edged swords of symbiotic rhizobia. Biochem J.

[11] Teulet A, Camuel A, Perret X, Giraud E (2022). The versatile roles of Type III secretion systems in rhizobium-legume symbioses. Annu Rev Microbiol.

[12] Teulet A, Busset N, Fardoux J, Gully D, Chaintreuil C, CArtieaux F (2019). The rhizobial type III effector ErnA confers the ability to form nodules in legumes. Proc Natl Acad Sci USA.

[13] Ratu STN, Teulet A, Miwa H, Masuda S, Nguyen HP, Yasuda M (2021). Rhizobia use a pathogenic-like effector to hijack leguminous nodulation signaling. Sci Rep..

[14] Ratu STN, Hirata A, Kalaw CO, Yasuda M, Tabuchi M, Okazaki S (2021). Multiple domains in the rhizobial type III effector Bel2-5 determine symbiotic efficiency with soybean. Front Plant Sci.

[15] Busset N, Gully D, Teulet A, Fardoux J, Camuel A, Cornu D (2021). The Type III effectome of the symbiotic *Bradyrhizobium vignae* strain ORS3257. Biomolecules.

[16] Vincent J. A manual for the practical study of root-nodule bacteria. Oxford, U.K: Blackwell Scientific Publications; 1970.

[17] Sadowsky MJ, Tully RE, Cregan PB, Keyser HH (1987). Genetic diversity in *Bradyrhizobium japonicum* serogroup 123 and its relation to genotype-specific nodulation of soybean. Appl Environ Microbiol.

[18] Gueye F, Moulin L, Sylla S, Ndoye I, Béna G (2009). Genetic diversity and distribution of *Bradyrhizobium* and *Azorhizobium* strains associated with the herb legume *Zornia glochidiata* sampled from across Senegal. Syst Appl Microbiol.

[19] Sambrook J, Fritsch EF, Maniatis TA. Molecular cloning: a laboratory manual, 2nd ed. Cold Spring Harbor, NY: Cold Spring Harbor Laboratory; 1989.

[20] Plener L, Manfredi P, Valls M, Genin S (2010). PrhG, a transcriptional regulator responding to growth conditions, is involved in the control of the type III secretion system regulon in *Ralstonia solanacearum*. J Bacteriol.

[21] Le Quéré A, Gully D, Teulet A, Navarro E, Gargani D, Fardoux J (2019). Complete genome sequence of *Bradyrhizobium* sp. strain ORS3257, an efficient nitrogen-fixing bacterium isolated from cowpea in Senegal. Microbiol Resour Announc.

[22] Orjuela J, Comte A, Ravel S, Charriat F, Vi T, Sabot F (2022). CulebrONT: a streamlined long reads multi-assembled pipeline for prokaryotic and eukaryotic genomes. Peer Comm J..

[23] Vallenet D, Belda E, Calteau A, Cruveiller S, Engelen S, Lajus A (2013). MicroScope-an integrated microbial resource for the curation and comparative analysis of genomic and metabolic data. Nucleic Acids Res.

[24] Nguyen LT, Schmidt HA, von Haeseler A, Minh BQ (2015). IQ-TREE: a fast and effective stochastic algorithm for estimating maximum-likelihood phylogenies. Mol Biol Evol.

[25] Gautreau G, Bazin A, Gachet M, Planel R, Burlot L, Dubois M (2020). PPanGGOLiN: depicting microbial diversity via a partitioned pangenome graph. PLoS Comput Biol.

[26] Kalyaanamoorthy S, Minh BQ, Wong TKF, von Haeseler A, Jermiin LS (2017). ModelFinder: fast model selection for accurate phylogenetic estimates. Nat Methods.

[27] Hoang D, Chernomor O, von Haeseler A, Minh BQ, Vinh LS (2018). UFBoot2: improving the ultrafast bootstrap approximation. Mol Biol Evol.

[28] Letunic I, Bork P (2021). Interactive tree of life (iTOL) v5: an online tool for phylogenetic tree display and annotation. Nucleic Acids Res.

[29] Sievers F, Barton GJ, Higgins DG. Multiple sequence alignment, vol 227. In: Baxevanis AD, Bader GD, Wishart DS, editors. Bioinformatics; Wiley; Hoboken, NJ, USA; 2020. p. 227–50.

[30] Wongdee J, Songwattana P, Nouwen N, Noisangiam R, Fardoux J, Chaintreuil C (2016). *nifDK* clusters located on the chromosome and megaplasmid of *Bradyrhizobium* sp. strain DOA9 contribute differently to nitrogenase activity during symbiosis and free- living growth. Mol Plant Microbe Interact.

[31] Tsai JW, Alley MR (2000). Proteolysis of the McpA chemoreceptor does not require the *Caulobacter* major chemotaxis operon. J Bacteriol.

[32] Oke V, Long SR (1999). Bacterial genes induced within the nodule during the Rhizobium- legume symbiosis. Mol Microbiol.

[33] Bonaldi K, Gourion B, Fardoux J, Hannibal L, Cartieaux F, Boursot M (2010). Large-scale transposon mutagenesis of photosynthetic *Bradyrhizobium* sp. strain ORS278 reveals new genetic loci putatively important for Nod-independent symbiosis with *Aeschynomene indica*. Mol Plant-Microbe Interact.

[34] Deslandes L, Olivier J, Peeters N, Feng DX, Khounlotham M, Boucher C (2003). Physical interaction between RRS1-R, a protein conferring resistance to bacterial wilt, and PopP2, a type III effector targeted to the plant nucleus. Proc Natl Acad Sci USA.

[35] Tasset C, Bernoux M, Jauneau A, Pouzet C, Brière C, Kieffer-Jacquinod S et al. Autoacetylation of the *Ralstonia solanacearum* effector PopP2 targets a lysine residue essential for RRS1-R-mediated immunity in *Arabidopsis*. PLoS Pathog. 2010. 10.1371/journal.ppat.1001202. 10.1371/journal.ppat.1001202 PMC2987829 21124938

[36] Lonjon F, Peeters N, Genin S, Vailleau F (2018). In vitro and in vivo secretion/translocation assays to identify novel *Ralstonia solanacearum* Type 3 Effectors. Methods Mol Biol.

[37] Mattanovich D, Rüker F, da Camara Machado, Laimer M, Regner F, Steinkellner H (1989). Efficient transformation of *Agrobacterium tumefaciens* by electroporation. Nucleic Acids Res.

[38] Bonaldi K, Gherbi H, Franche C, Bastien G, Fardoux J, Barker D (2010). The Nod factor-independent symbiotic signaling pathway: development of *Agrobacterium rhizogenes*- mediated transformation for the legume *Aeschynomene indica*. Mol Plant Microbe Interact.

[39] Czernic P, Gully D, Cartieaux F, Moulin L, Guefrachi I, Patrel D (2015). Convergent evolution of endosymbiont differentiation in Dalbergioid and inverted repeat-lacking clade legumes mediated by nodule-specific cysteine-rich peptides. Plant Physiol.

[40] Avontuur JR, Palmer M, Beukes CW, Chan WY, Coetzee MPA, Blom J (2019). Genome-informed *Bradyrhizobium* taxonomy: where to from here?. Syst Appl Microbiol.

[41] Teulet A, Gully D, Rouy Z, Camuel A, Koebnik R, Giraud E (2020). Phylogenetic distribution and evolutionary dynamics of *nod* and T3SS genes in the genus *Bradyrhizobium*. Micro Genom.

[42] Cunnac S, Occhialini A, Barberis P, Boucher C, Genin S (2004). Inventory and functional analysis of the large Hrp regulon in *Ralstonia solanacearum:* identification of novel effector proteins translocated to plant host cells through the type III secretion system. Mol Microbiol.

[43] Sperschneider J, Catanzariti AM, DeBoer K, Petre B, Gardiner DM, Singh KB (2017). LOCALIZER: subcellular localization prediction of both plant and effector proteins in the plant cell. Sci Rep.

[44] Bailey TL, Johnson J, Grant CE, Noble WS (2015). “The MEME Suite”. Nucleic Acids Res.

[45] Hwang S, Gou G, Kuznetsov IB (2007). DP-Bind: a web server for sequence-based prediction of DNA-binding residues in DNA-binding proteins. Bioinformatics.

[46] Zhao Q, Xie Y, Zheng Y, Jiang S, Liu W, Mu W (2014). GPS-SUMO: A tool for the prediction of sumoylation sites and SUMO-interaction motifs. Nucleic Acids Res.

[47] Kerscher O (2007). SUMO junction-what’s your function? New insights through SUMO-interacting motifs. EMBO Rep.

[48] Miché L, Moulin L, Chaintreuil C, Contreras-Jimenez JL, Munive-Hernández JA, Del Carmen Villegas-Hernandez M (2010). Diversity analyses of *Aeschynomene* symbionts in tropical Africa and central America reveal that nod-independent stem nodulation is not restricted to photosynthetic bradyrhizobia. Environ Microbiol.

[49] Giraud E, Hannibal L, Fardoux J, Verméglio A, Dreyfus B (2000). Effect of *Bradyrhizobium* photosynthesis on stem nodulation of *Aeschynomene sensitiva*. Proc Natl Acad Sci USA.

[50] Quilbé J, Lamy L, Brottier L, Leleux P, Fardoux J, Rivallan R (2021). Genetics of nodulation in *Aeschynomene evenia* uncovers mechanisms of the rhizobium-legume symbiosis. Nat Commun.

[51] Gibson KE, Kobayashi H, Walker GC (2008). Molecular determinants of a symbiotic chronic infection. Annu Rev Genet.

[52] Janczarek M, Rachwał K, Marzec A, Grządziel J, Palusińska-Szysz M (2015). Signal molecules and cell-surface components involved in early stages of the legume-rhizobium interactions. Appl Soil Ecol.

[53] Poole P, Ramachandran V, Terpolilli J (2018). Rhizobia: from saprophytes to endosymbionts. Nat Rev Microbiol.

[54] Johnson ES (2004). Protein modification by SUMO. Ann Rev Biochem.

[55] Augustine RC, Vierstra RD (2018). SUMOylation: re-wiring the plant nucleus during stress development. Curr Opin Plant Biol.

[56] Morrell R, Sadanandom A (2019). Dealing with stress: a review of plant SUMO proteases. Front Plant Sci.

[57] Kim JG, Stork W, Mudgett MB (2013). *Xanthomonas* type III effector XopD desumoylates tomato transcription factor SlERF4 to suppress ethylene responses and promote pathogen growth. Cell Host Microbe.

[58] Yau TY, Sander W, Eidson C, Courey AJ (2021). SUMO interacting motifs: structure and function. cells.

[59] Tria FDK, Landan G, Dagan T (2017). Phylogenetic rooting using minimal ancestor deviation. Nat Ecol Evol.

[60] Sanchez C, Mercante V, Babuin MF, Lepek VC (2012). Dual effect of *Mesorhizobium loti* T3SS functionality on the symbiotic process. FEMS Microbiol Lett.

[61] Rodrigues JA, Lopez-Baena FJ, Ollero FJ, Vinardell JM, Espuny MR, Bellogin RA (2007). NopM and NopD are rhizobial nodulation outer proteins: identification using LC-MALDI and LC-ESI with a monolithic capillary column. J Proteome Res.

[62] Okazaki S, Zehner S, Hempel J, Lang K, Göttfert M (2009). Genetic organization and functional analysis of the type III secretion system of *Bradyrhizobium elkanii*. FEMS Microbiol Lett.

[63] Faruque OM, Miwa H, Yasuda M, Fujii Y, Kaneko T, Sato S (2015). Identification of *Bradyrhizobium elkanii* genes involved in incompatibility with soybean plants carrying the Rj4 Allele. Appl Environ Microbiol.

