# Supplementary material for: Widespread Bradyrhizobium distribution of diverse Type III effectors that trigger legume nodulation in the absence of Nod factor
Source: ISME J. 2023 Jun 24;17(9):1416–29. doi: 10.1038/s41396-023-01458-1 (PMC10432411; doi:10.1038/s41396-023-01458-1)
Supplement: Supplementary file 1 — Figure S1 [file 41396_2023_1458_MOESM1_ESM.pdf]

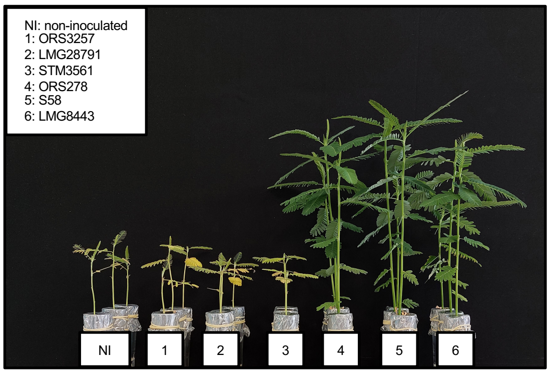

Figure S1. The NF-independent T3SS-dependent symbiosis is less efficient for fixing nitrogen than the NF-independent T3SS-independent symbiosis in *A. indica*.
Comparison of the growth of the plant (leaf phenotype) non-inoculated (NI), inoculated with strains able to fix nitrogen with a low efficiency in a T3SS-dependent manner (Type E): ORS3257 (1), LMG28791 (2) and STM3561 (3) ; or with strains belonging to photosynthetic supergroup able to fix efficiently nitrogen in a T3SS-independent manner (Type F): ORS278 (4), S58 (5) and LMG8443 (6). Pictures were taken 21 days after inoculation.
