# Supplementary material for: Widespread Bradyrhizobium distribution of diverse Type III effectors that trigger legume nodulation in the absence of Nod factor
Source: ISME J. 2023 Jun 24;17(9):1416–29. doi: 10.1038/s41396-023-01458-1 (PMC10432411; doi:10.1038/s41396-023-01458-1)
Supplement: Supplementary file 2 — Figure S2 [file 41396_2023_1458_MOESM2_ESM.pdf]

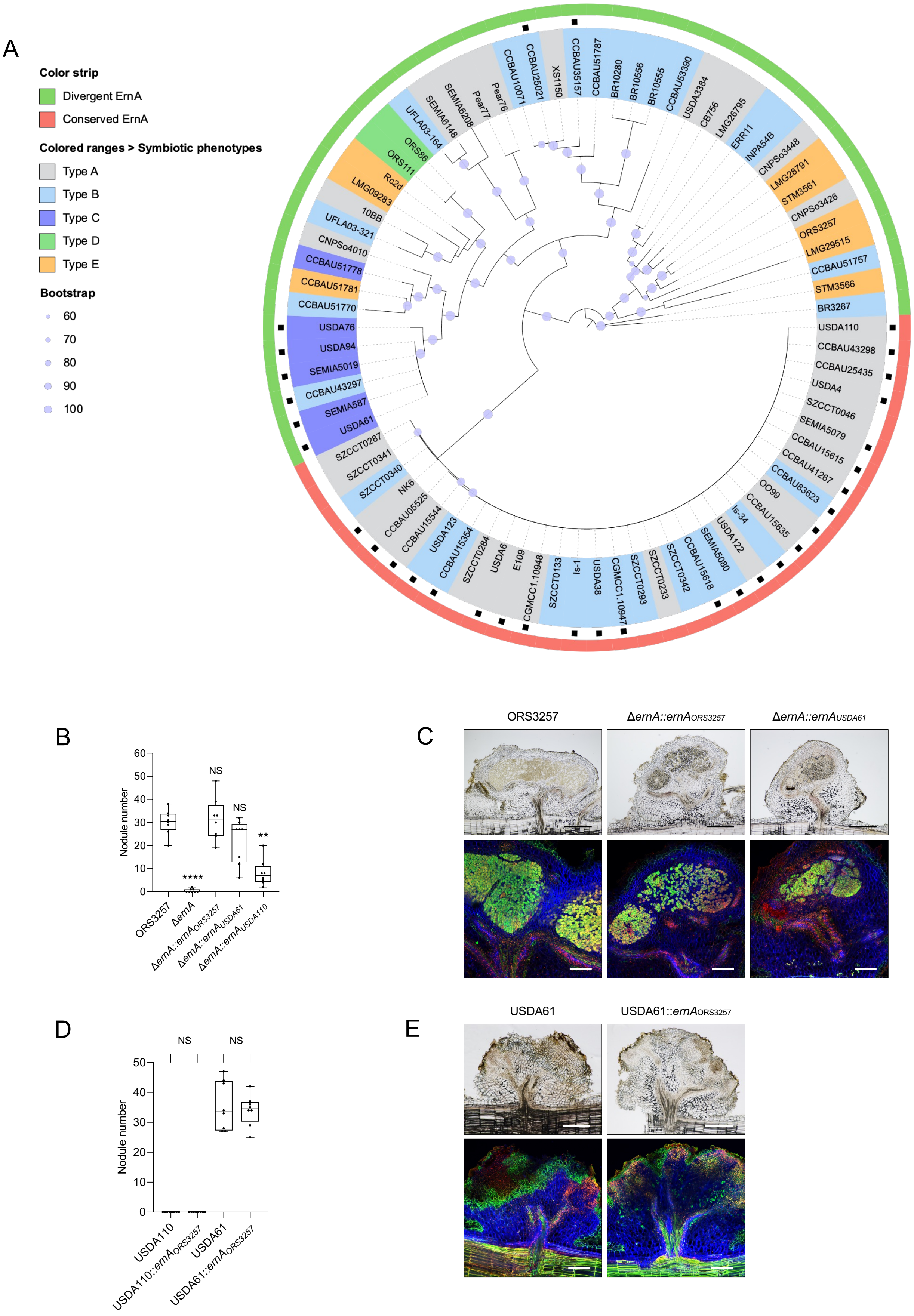

Figure S2. Exploring the natural variability of ErnA on nodulation capacity and infection.

(A). Phylogenetic tree of full-length ErnA homologs identified in the collection of bradyrhizobia used in this study. Two main clusters are identified and indicated by colour strip according to their level of conservation. The symbiotic phenotype of each strain having an ErnA homolog is indicated by a coloured range referenced in the key. The black squares indicate strains isolated from soybean nodule. (B and D). Nodule number on *A. indica* plants at 21 days after inoculation with ORS3257 (B) or USDA61 and USDA110 (D) and their respective mutants containing various *ernA* genes. Box plots show the results of one of the two experiments performed independently (8 plants each). ** $p \leq 0.01$, **** $p < 0.0001$ significant differences between the wild-type strain ORS3257 and each mutant using a parametric Welch test, NS: not significant. No significant differences were calculated between the wild-type strain USDA61 and USDA110 with their respective mutant (D) using a nonparametric Kruskal-Wallis test, NS: not significant. (C and E) Cross-sections of nodules were observed by light or confocal microscopy after staining with SYTO 9, propidium iodide and calcofluor; scale bars C and E, 200 µm. Intracellular infection is visible in C while only intercellular infection can sometimes be observed in E.
