# Supplementary material for: Widespread Bradyrhizobium distribution of diverse Type III effectors that trigger legume nodulation in the absence of Nod factor
Source: ISME J. 2023 Jun 24;17(9):1416–29. doi: 10.1038/s41396-023-01458-1 (PMC10432411; doi:10.1038/s41396-023-01458-1)
Supplement: Supplementary file 3 — Figure S3 [file 41396_2023_1458_MOESM3_ESM.pdf]

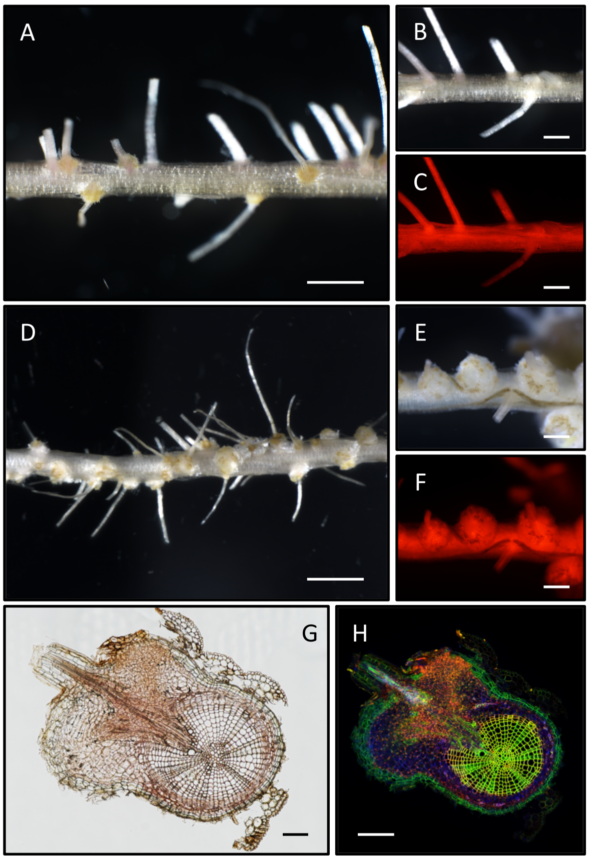

Figure S3. Ectopic expression of *sup3* in *A. indica* roots induces spontaneous nodules.
(A-F) *A. indica* roots transformed either with the empty vector containing the DsRed marker (A-C) or with *p35S-sup3* (D-F) at 7 weeks after transformation and in the absence of bradyrhizobia. Roots were observed by a light and fluorescence stereomicroscope equipped with a DsRed filter. (Scale bars: A and D, 2 mm; B, C, E and F, 500 µm). Pseudo-nodules have peripheral vascularisation (G and H). Cross-sections of pseudo-nodules were observed by light or confocal microscopy after staining with SYTO 9, propidium iodide and calcofluor; scale bars G and H, 200 µm. No bacteria were observed in the root tissues.
