# Supplementary material for: Widespread Bradyrhizobium distribution of diverse Type III effectors that trigger legume nodulation in the absence of Nod factor
Source: ISME J. 2023 Jun 24;17(9):1416–29. doi: 10.1038/s41396-023-01458-1 (PMC10432411; doi:10.1038/s41396-023-01458-1)
Supplement: Supplementary file 4 — Figure S4 [file 41396_2023_1458_MOESM4_ESM.pdf]

A

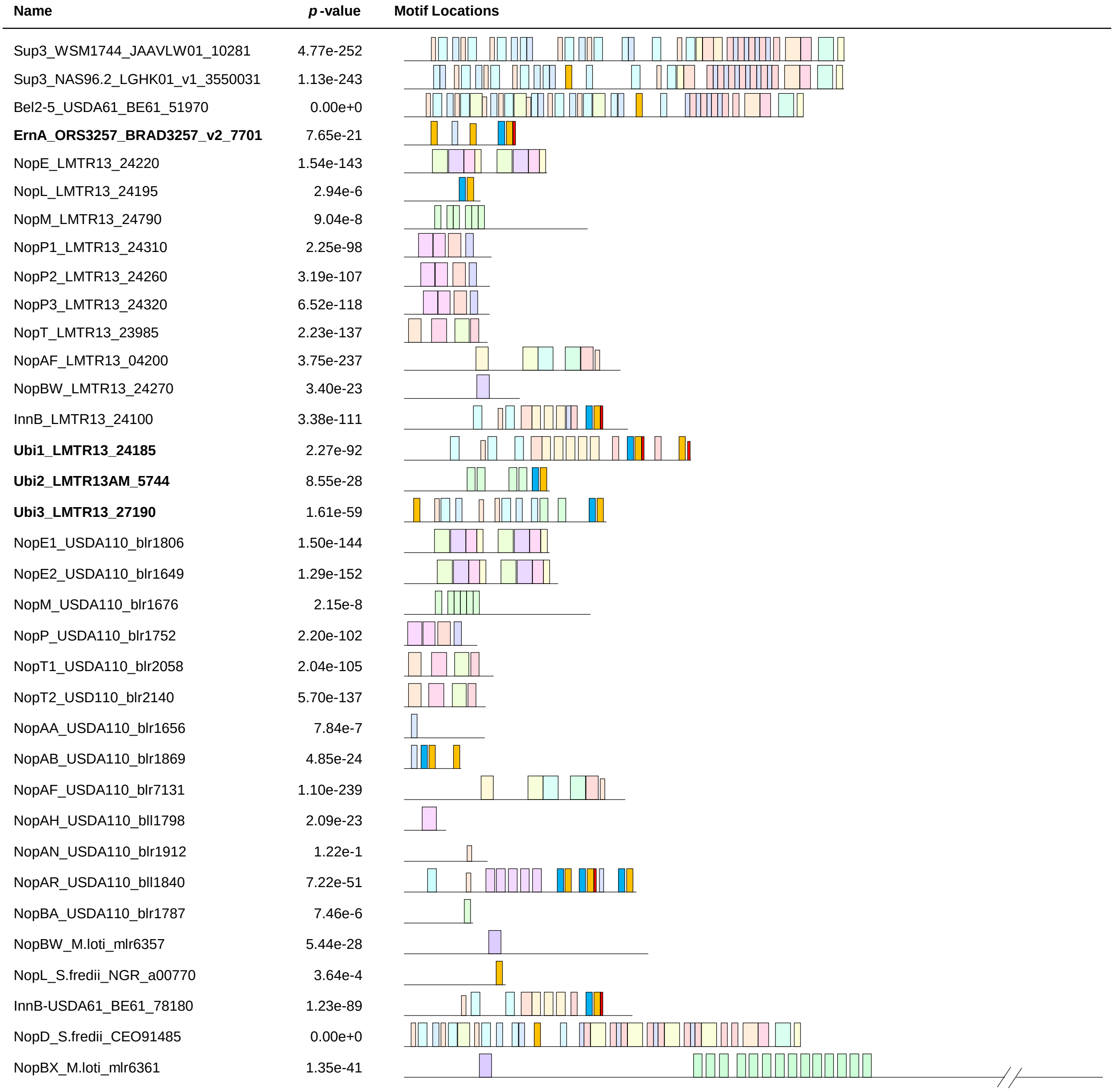

B

Motif	Symbol	Motif Consensus
1.		FVDLNAPTPSELRDDAHFAPA
2.		TARNYATALRSFSRWLRANNKPSIAARLN
3.		VWSHEVKDGKATINLGDKYTITADEKDGTWTVRNNQTGHVSKIHGDPHVD
4.		GKDDFDFKKGMTFQLDDGTKITVDTVDYGKGKTISSKLTITNGDNAMVVE
5.		PSSFQELPATPATPSAGAWDWFREQMQEP
6.		GFBPQHLANLVNGFSKWPEEENSRQATIA
7.		PPARARSDTYGGLES
8.		LGDDKDGKNNLKVTQSNAGRTLDZLTSDGAQTIHEQ
9.		LYPEDAALISGLKEA
10.		FPPPFAGPVPGHHQGARQLGSPQGLSPVSAHSDDDALAWLSEELARQMQE
11.		LPAGLRRLBVDNNQLTSLPET
12.		VAYHYDSYGGHNHEAAAQLARRLNLRLESPGMAQQQNSYDCGVFVVDGTR
13.		RLLAALDHLRTSQSTGGVTVI
**14.**		**PPTQFLINGERYTAELGPGGR**
15.		YSLVSEPPVVEIDKSSFERELRDFYGDDIEDIAANPQEYSDFVSEKA
16.		LRFGAESDVLRAFQRIVYDPNGNDTADFLFLPVND
17.		GRTEJTSVVDLRVTHPLVENAGDILLEHQLRLDGERPLJLSR
18.		GHHGLELGDTEWLGDEHIQRDYELQEQDLQRDDPDLAARTRFVDPLIAHY
19.		GQGWVDGAGRQVNQASIDAGE
20.		LDEDVESYKKGAGADSRIGAALAQLRKSQAGAKAMEPERH
**21.**		**WSHGSQPAPDALIDILGNIGL**
22.		VDPEHFTFDPVQFPPGELRRLLDDEP
23.		RFNFPNKFLVYQHDTPDKNLFAKEERAFSHGCMRVQNPDQYASVLLNIVM
24.		VMQRGQPVWTTRVVTGKPGKHATPLLTETMKFITVNPTWNVPPSIIYNEY
25.		AAQHSASQGAVSWPEVLPEGD
26.		QDLFLGLMDEPGPSSSLZP
27.		MGFVEVDDNHMVLDPSQHPDKWTKN
28.		CVEWWDKKVQRHERMRAVCVATABLPSSTAEMRQEEEAALR
29.		TFBAPVPWPTMSPTVHSPVQSVPQDALFD
30.		TVQVAYAYGTVQDSEQARYFSYQLGDKSVGLLRTEGGVRM
31.		LFEYRTAELPQANVDGICVGLTAEWLRNLSNSPSTRMGALLPGSESHASA
32.		HTIATSASNGTTTLFDPNYGEFTVRSD
33.		EKYTPERIRSMYGKSEIDLKFPTPIPVNITYQTAFVDDAGK
34.		DLLRLRRYQQLVEANRALGLAPPEQAGSPAAQGARQ
35.		DEPSQSVDSGSFTETLADLAPQWSLPPGELPDKMGACCSKP
**36.**		**DVRLIHHP**
37.		ELKLTASMFDYARQAQSGRMHWSQVAADILYPEHPVDPAEV
38.		EQLNLDNVVVNRQALQARLRG
39.		VKAJNSPKRDKQIDVVLVNMERWRWLPRDLGVPSLGDAYVILNIPDFTLK
40.		QNTILQEAGLEPSGEEKRYAFGESSSIDKMVNEITEDGSNHLLSLYF

C

Sup3_WSM1744_JAAVLW01_10	Sup3_NAS96.2_LGHK01_v1_3
Bel2-5_USDA61_BE61_51970	ErnA_ORS3257_BRAD3257_v2
NopE_LMTR13_24220	NopH_LMTR13_24225
NopC_LMTR13_24215	NopL_LMTR13_24195
NopM_LMTR13_24790	NopP1_LMTR13_24310
NopP2_LMTR13_24260	NopP3_LMTR13_24320
NopT_LMTR13_23985	NopAF_LMTR13_04200
NopAJ_LMTR13AM_6340	NopBW_LMTR13_24270
InnB_LMTR13_24100	Ubi1_LMTR13_24185
Ubi2_LMTR13AM_5744	Ubi3_LMTR13_27190
NopH_USDA110_bll1804	NopE1_USDA110_blr1806
NopE2_USDA110_blr1649	NopM_USDA110_blr1676
NopP_USDA110_blr1752	NopT1_USDA110_blr2058
NopT2_USD110_blr2140	NopAA_USDA110_blr1656
NopAB_USDA110_blr1869	NopAC_USDA110_blr1993
NopAF_USDA110_blr7131	NopAG_USDA110_bll1862
NopAK_USDA110_blr1789	NopAJ_USDA110_bll1846
NopAH_USDA110_bll1798	NopAI_USDA110_bll1796
NopAN_USDA110_blr1912	NopAR_USDA110_bll1840
NopAS_USDA110_blr1626	NopAZ_USDA110_blr0077
NopBA_USDA110_blr1787	NopBK_USDA110_blr1975
NopX_S.fredii_NGR_a00700	NopBW_M.loti_mlr6358
NopL_S.fredii_NGR_a00770	NopJ_S.fredii_NGR_a02610
NopC_S.fredii_AAY33494	InnB-USDA61_BE61_78180
NopD_S.fredii_CEO91485	NopBX_M.loti_mlr6361

Figure S4. MEME analysis on putative ET-Nods and functionally characterized T3Es.
(A). Schematic representation of the conserved motifs (40) identified in an input of 50 rhizobial T3Es using the MEME suite tools [44]. This input includes the 13 putative T3Es identified in LMTR13 and 37 rhizobial T3Es previously characterized [10,12]. The parameters used for the MEME analysis were the following : i) The maximum number of motif identification was restricted to 40, ii) The occurrence of motif was set to any number of repetitions and iii) the minimum motif width was set to 6 and a maximum of 50. The panel A displays only the 35 T3Es in which conserved motives were identified by MEME. The ID of the protein, the p-value and the motif locations identified into the protein appears in column 1, 2 and 3 respectively. ErnA, Ubi1, Ubi2 and Ubi3 are shown in bold. (B). Corresponding colour and consensus of each motif. M1, M2 and M3 conserved motifs appear in bold, respectively in the 14th, 21st and 36th position. (C). Complete list of the T3Es used as input.
